# Safe Endoscopic Treatment for a Bleeding Infant With Multifocal Lymphangioendotheliomatosis and Thrombocytopenia (MLT)

**DOI:** 10.14309/crj.0000000000001755

**Published:** 2025-06-27

**Authors:** Or Steg Saban, Elena Pope, Govind B. Chavhan, Manuel Carcao, Susanna Talarico, Simon C. Ling

**Affiliations:** 1 Division of Gastroenterology, Hepatology and Nutrition, The Hospital for Sick Children, Toronto, ON; 2 Division of Pediatric Dermatology, The Hospital for Sick Children, Toronto, ON; 3 Diagnostic and Interventional Radiology, The Hospital for Sick Children, Toronto, ON; 4 Division of Haematology/Oncology, The Hospital for Sick Children, Toronto, ON; 5 Division of Pediatric Medicine, The Hospital for Sick Children, Toronto, ON

**Keywords:** MLT, GI bleeding, hematemesis, cutaneous lesions

## Abstract

We present the case of a 3-week-old girl with multiple cutaneous vascular lesions, melena, hematemesis, severe anemia (hemoglobin 4.4 g/dL), and thrombocytopenia (72 × 10^3^/µL), with clinical features consistent with multifocal lymphangioendotheliomatosis with thrombocytopenia (MLT). Upper gastrointestinal endoscopy revealed numerous actively bleeding angiomatous lesions, measuring 1–3 mm in diameter. Homeostasis was successfully achieved using argon plasma coagulation (APC). To our knowledge, this is among the few reports describing the effective use of APC in managing life-threatening gastric hemorrhage in an infant with MLT. While gastrointestinal perforation has been previously reported as a complication of endoscopic therapy in this context, our findings suggest that APC may be a viable option for refractory bleeding, provided that surgical support is immediately accessible.

## INTRODUCTION

The evaluation and management of significant upper gastrointestinal (GI) bleeding in neonates present unique challenges, particularly due to limitations in endoscopic instrumentation and the technical difficulties associated with small patient size. The neonatal endoscope, with a 5.5 mm shared suction and working channel, often proves inadequate for effective aspiration of blood clots and accommodates only a limited range of therapeutic tools—specifically a sclerotherapy needle and an argon plasma coagulation (APC) catheter. While a standard pediatric endoscope (8.8 mm diameter) may be used in select neonates to improve diagnostic and therapeutic capabilities, the underlying etiology of bleeding frequently remains unidentified.

In a retrospective review of 56 infants aged younger than 12 months who presented with GI bleeding, a definitive source was identified by endoscopy in only 12.5% (7/56) of cases. These included esophageal varices, peptic ulcers, angiodysplasia, and anastomotic ulcers.^1^ Among those, only 3 infants underwent endoscopic therapy (eg, variceal banding or sclerotherapy, and triamcinolone injection). Notably, 3 infants (5.3%) developed gastric or jejunal perforations shortly after the procedure. These findings underscore the limited diagnostic yield and significant procedural risks associated with neonatal endoscopy in the setting of major GI bleeding.

## CASE REPORT

**Figure 1.**
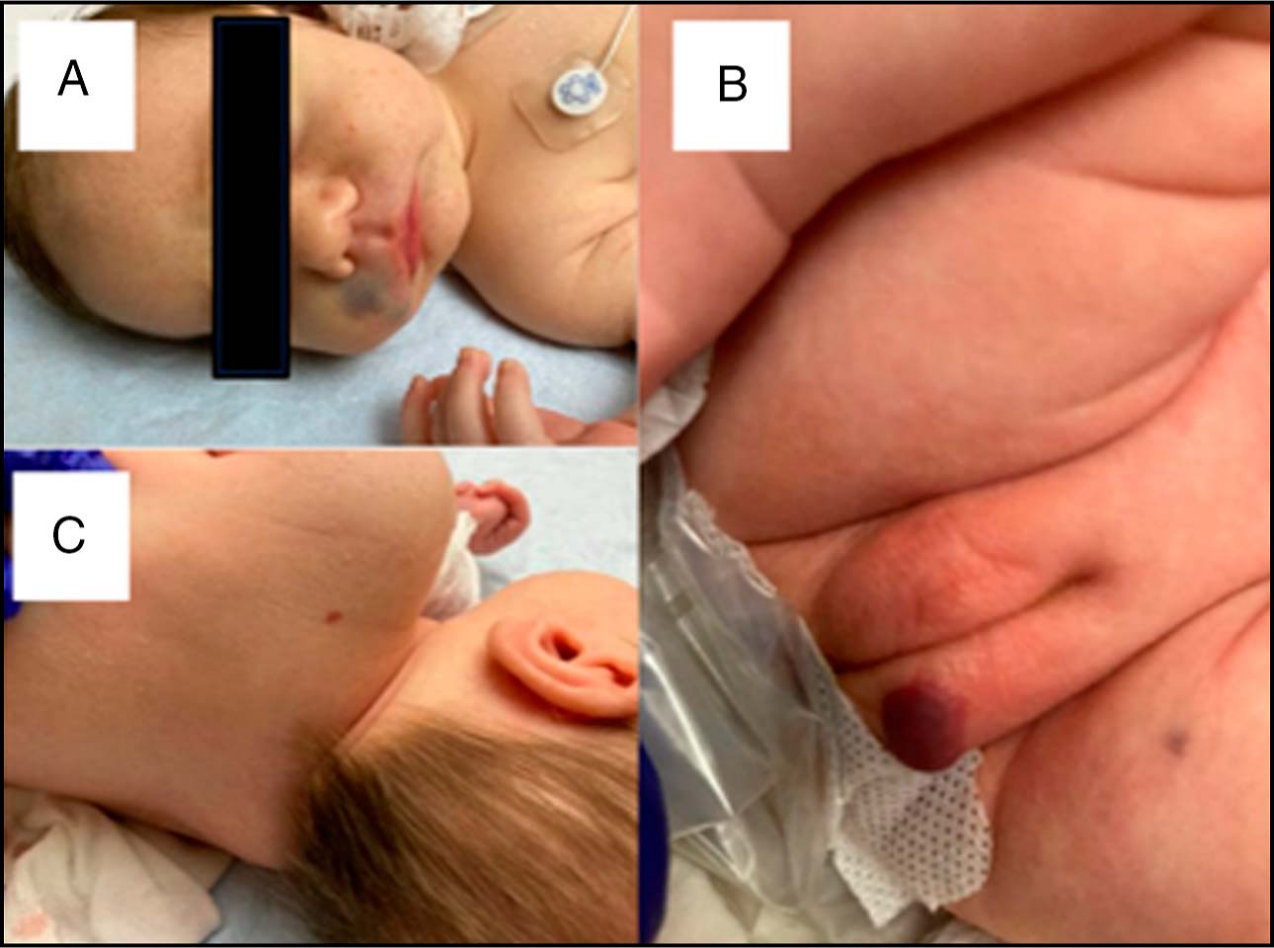
Cutaneous lesions observed. Ecchymosis on the upper lip (A), large erythematous plaque on the labia majora (B), and a bright red papule on the back (C).

A 3-week-old female infant was transferred to our tertiary care center from a community hospital due to hematemesis and melena. Her prenatal, perinatal, and family histories were unremarkable, and she had received standard vitamin K prophylaxis at birth. On admission, she appeared pale, weighed 3.8 kg, and was tachycardic. Cutaneous examination revealed multiple vascular lesions, including ecchymosis on the upper lip and thigh, a large erythematous plaque on the labia majora, and numerous red papules distributed across the back, limbs, and right upper eyelid (Figure 1).

Laboratory evaluation showed a hemoglobin level of 10 g/dL (a significant drop from 21.5 g/dL at birth) and thrombocytopenia (72 × 10^3^/μL). Liver biochemistry, international normalized ratio, and partial thromboplastin time were within normal limits. Abdominal ultrasonography was unremarkable, showing normal hepatic parenchyma and portal vein architecture without evidence of internal hemangiomas, masses, or splenomegaly.

**Figure 2.**
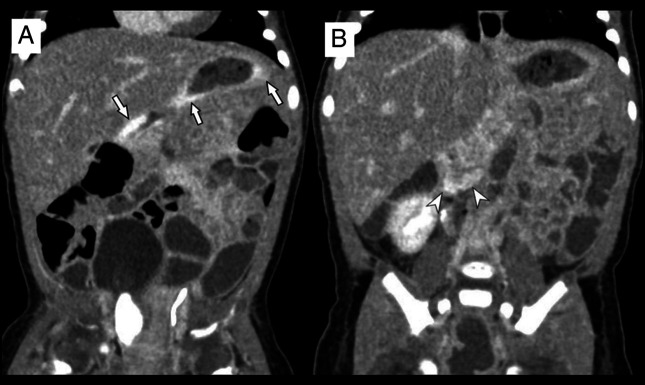
Abdominal computed tomography images in coronal plane showed multiple enhancing lesions in the gastric mucosa (arrows, A) and contrast extravasation in the gastric antrum (B).

Contrast-enhanced abdominal computed tomography revealed multiple enhancing lesions within the gastric mucosa, with contrast extravasation into the gastric lumen during the venous phase (Figure 2). Magnetic resonance imaging identified multiple small, irregular T2 hyperintense lesions involving the metaphyseal margins of the long bones, pelvis, and ribs, as well as subcutaneous and muscular tissues.

Despite intravenous pantoprazole, the patient continued to experience significant GI bleeding, with hemoglobin dropping to 4.4 g/dL, necessitating multiple red blood cell and platelet transfusions and intensive care unit admission. The combination of vascular skin lesions, bone involvement, severe upper GI bleeding, and thrombocytopenia raised suspicion for a visceral vascular anomaly.^2,3^ The multifocal nature of the lesions, associated thrombocytopenia, and mixed lesion morphology were most consistent with multifocal lymphangioendotheliomatosis with thrombocytopenia (MLT), also known as cutaneovisceral angiomatosis with thrombocytopenia.^2,4,5^

MLT is primarily a clinical diagnosis. Although immunohistochemical staining for lymphatic vessel endothelial hyaluronan receptor 1 can support the diagnosis, this test is not routinely available in clinical practice.^4^

**Figure 3.**
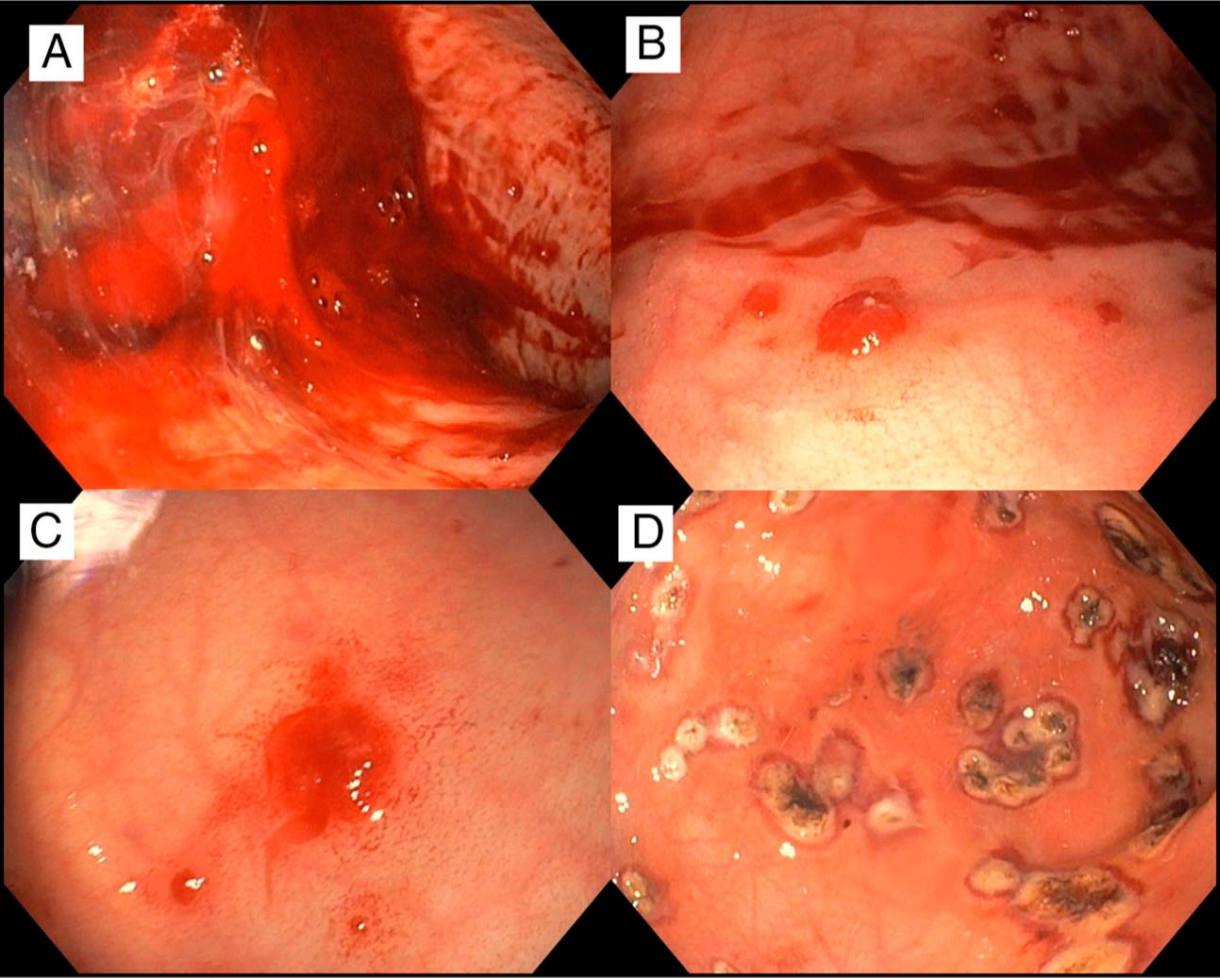
Endoscopy findings. Active bleeding was observed in the stomach (A), with multiple angiomatous raised lesions oozing (B and C). After argon plasma coagulation was performed, bleeding ceased (D).

Given ongoing severe bleeding, upper GI endoscopy was performed using a pediatric gastroscope (GIF-H190). APC was administered through a FiAPC probe (diameter 1.5–2.3 mm) at settings of 1.2 L/min and 2 W maximum. Endoscopy revealed multiple raised, actively bleeding vascular lesions in the gastric mucosa (Figure 3), all of which were successfully treated with APC without complications.

The patient was initiated on oral sirolimus at 0.025 mg/kg/dose twice daily, with a target trough level of 5–15 µg/L, and prophylactic trimethoprim-sulfamethoxazole for *Pneumocystis jirovecii* pneumonia prevention.^6–8^

At the age of 9 months, the patient experienced a second episode of hematemesis. Repeat endoscopy again demonstrated multiple actively bleeding gastric vascular lesions, which were successfully treated with APC. In view of the recurrent bleeding and multifocal nature of the lesions, we instituted a scheduled surveillance and prophylactic endoscopy program at intervals of 3 to 6 months to reduce the risk of acute bleeding events and avoid urgent procedures requiring specialized personnel.

**Figure 4.**
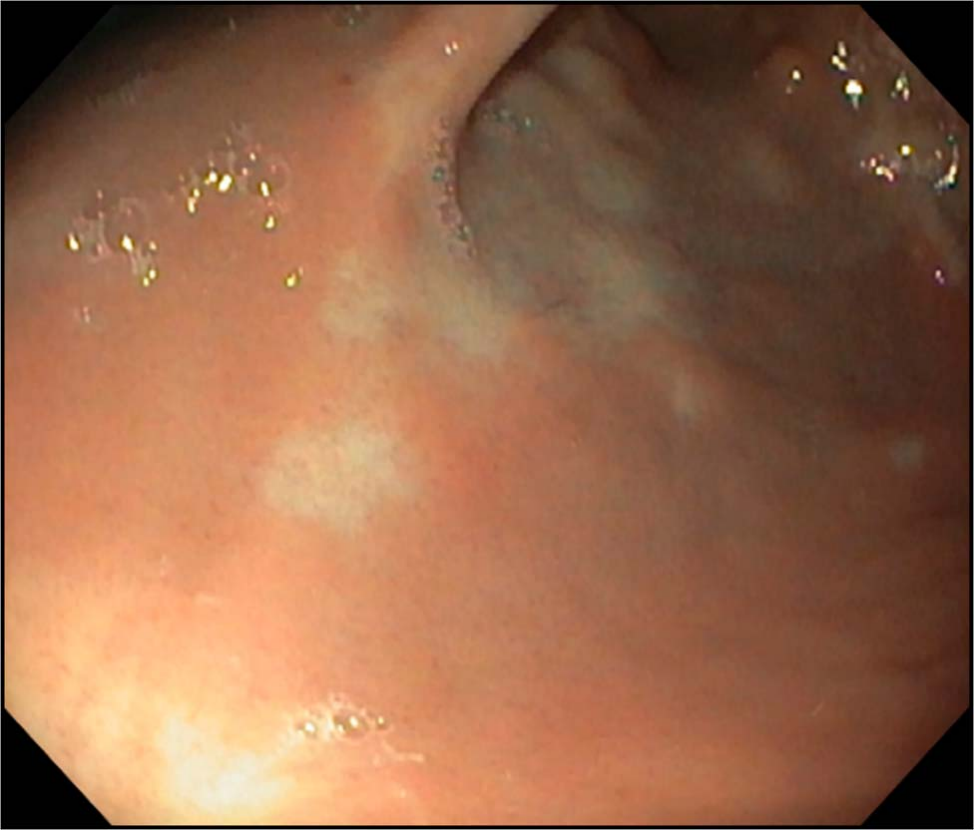
In the last endoscopy performed, at the age of 34 months, no lesions were observed and no active bleeding. Postargon plasma coagulation scars are seen.

Since diagnosis, the patient has undergone 6 upper endoscopies: 3 in response to overt GI bleeding (melena or hematemesis) and 3 electively. APC was used in all procedures to treat both actively bleeding and visible non-bleeding lesions in an effort to prevent progression or future hemorrhage. Following her third endoscopy at the age of 13 months, no further bleeding episodes were reported. Her most recent endoscopy, performed at nearly the age of 3 years, showed healed APC treatment sites with only a single residual small lesion (Figure 4).

## DISCUSSION

MLT is a rare congenital vascular disorder characterized by the presence of multiple cutaneous and visceral vascular lesions in association with thrombocytopenia. Patients typically present within the first few weeks of life, most commonly with upper GI bleeding, due to mucosal vascular lesions throughout the GI tract. Lesions may also involve other organ systems including the brain, bones, kidneys, and soft tissues. The thrombocytopenia and coagulopathy observed in MLT are attributed to chronic intravascular coagulation and consumption of platelets and clotting factors within these abnormal vascular channels. Histologically, MLT lesions consist of elongated, thin-walled vessels lined by a single layer of endothelial cells with papillary intraluminal projections.

Management of significant GI bleeding in neonates is inherently challenging due to technical limitations in endoscopic equipment and concerns regarding procedural safety. The utility of endoscopy in this population is limited both diagnostically and therapeutically. Among 39 reported cases of MLT in the literature, 30 patients (77%) experienced GI bleeding during the disease course.^2,9,10^ However, the majority of these cases (26/30, 87%) were managed conservatively without endoscopic intervention. Notably, in a small series of 4 infants with MLT who underwent endoscopic treatment with APC, 3 experienced postprocedure gastric or intestinal perforations.^11^ Two of these infants (aged 13 days and 1 month) developed surgically confirmed gastric perforations at sites treated with APC. In another case, repeated endoscopic APC over a 4-month period failed to achieve sustained hemostasis, ultimately requiring surgical resection. A fourth infant underwent approximately 20 APC treatments but developed distal small bowel perforation at a site not directly treated. These reports raise concern regarding the safety of APC in neonates, particularly in the context of MLT, although it remains unclear whether the risk of perforation is primarily due to the underlying disease pathology, the technical aspects of endoscopy in neonates, or a combination of both. Furthermore, the transmural extent of these lesions is unknown, as there are no pathological studies of surgical resection specimens to assess this.

Despite these concerns, the decision to use APC in our patient was guided by the need for rapid hemostasis and the feasibility of treating multiple lesions in a single session. Compared with sclerotherapy, APC offers broader applicability for multifocal mucosal bleeding. Surgical options were considered but deemed high risk, given the number and distribution of lesions, many of which would not be visualized or accessible during laparotomy, and would require extensive wedge resections with significant morbidity.

This case highlights the successful use of APC to control severe upper GI bleeding in an infant with MLT, without complications, and in conjunction with sirolimus therapy. While APC has been associated with a risk of perforation, our experience supports its cautious use in select cases of refractory, life-threatening bleeding, particularly when performed by experienced endoscopists with immediate surgical backup available. Moreover, we demonstrate the potential benefit of routine, prophylactic APC treatments to prevent lesion progression and acute rebleeding events during the course of disease, especially while awaiting therapeutic response to medical management.

Given the rarity of MLT and the lack of consensus on optimal management, further case reports and prospective studies are needed to inform best practices and improve outcomes in this vulnerable patient population.

## DISCLOSURES

Author contributions: OS Saban: idea conception, writing; S. Talarico: reviewing; M. Carcao: reviewing; GB Chavhan: reviewing, finding imaging; E. Pope: establishing the diagnosis, reviewing; SC Ling: reviewing, writing. OS Saban is the article guarantor.

Financial disclosure: This research is supported by TRIANGLE Canada Training Program, the Canadian Institutes of Health Research, Canadian Association for the Study of the Liver, and the Canadian Association of Gastroenterology.

Previous presentation: Presented at the 55th Annual Meeting of the European Society for Paediatric Gastroenterology, Hepatology and Nutrition (ESPGHAN); May 17–20, 2023; Vienna, Austria.

Informed consent was obtained for this case report.

## ABBREVIATIONS:

APC: argon plasma coagulation
GI: gastrointestinal
MLT: multifocal lymphangiomatosis and thrombocytopenia.
